# Prospective, multicentre study of screening, investigation and management of hyponatraemia after subarachnoid haemorrhage in the UK and Ireland

**DOI:** 10.1136/svn-2022-001583

**Published:** 2022-09-23

**Authors:** James J M Loan, Steven Tominey, Kirun Baweja, Julie Woodfield, Thomas J G Chambers, Mark Haley, Simran S Kundu, H Y Josephine Tang, Anthony N Wiggins, Michael T C Poon, Paul M Brennan, the Sodium after Subarachnoid Haemorrhage (SaSH) audit collaborators, Midhun Mohan

**Affiliations:** 1 Translational Neurosurgery, The University of Edinburgh Centre for Clinical Brain Sciences, Edinburgh, UK; 2 Neurosurgery, NHS Lothian, Edinburgh, UK; 3 Neurosurgery, Institute of Neurological Sciences, Glasgow, UK; 4 Department of Medicine, School of Medicine, Dentisty and Nuring, University of Glasgow, Glasgow, UK; 5 Internal Medicine, Queen's University, Kingston, Ontario, Canada; 6 Centre for Discovery Brain Sciences, University of Edinburgh, Edinburgh, UK; 7 Edinburgh Centre for Diabetes and Endocrinology, NHS Lothian, Edinburgh, UK; 8 Radiology, University Hospitals Plymouth NHS Trust, Plymouth, UK; 9 Department of Medicine, Cork University Hospital, University College Cork, Cork, Ireland

**Keywords:** arteriovenous malformations, subarachnoid, hemorrhage, intracranial aneurysm, prospective studies

## Abstract

**Background:**

Hyponatraemia often occurs after subarachnoid haemorrhage (SAH). However, its clinical significance and optimal management are uncertain. We audited the screening, investigation and management of hyponatraemia after SAH.

**Methods:**

We prospectively identified consecutive patients with spontaneous SAH admitted to neurosurgical units in the United Kingdom or Ireland. We reviewed medical records daily from admission to discharge, 21 days or death and extracted all measurements of serum sodium to identify hyponatraemia (<135 mmol/L). Main outcomes were death/dependency at discharge or 21 days and admission duration >10 days. Associations of hyponatraemia with outcome were assessed using logistic regression with adjustment for predictors of outcome after SAH and admission duration. We assessed hyponatraemia-free survival using multivariable Cox regression.

**Results:**

175/407 (43%) patients admitted to 24 neurosurgical units developed hyponatraemia. 5976 serum sodium measurements were made. Serum osmolality, urine osmolality and urine sodium were measured in 30/166 (18%) hyponatraemic patients with complete data. The most frequently target daily fluid intake was >3 L and this did not differ during hyponatraemic or non-hyponatraemic episodes. 26% (n/N=42/164) patients with hyponatraemia received sodium supplementation. 133 (35%) patients were dead or dependent within the study period and 240 (68%) patients had hospital admission for over 10 days. In the multivariable analyses, hyponatraemia was associated with less dependency (adjusted OR (aOR)=0.35 (95% CI 0.17 to 0.69)) but longer admissions (aOR=3.2 (1.8 to 5.7)). World Federation of Neurosurgical Societies grade I–III, modified Fisher 2–4 and posterior circulation aneurysms were associated with greater hazards of hyponatraemia.

**Conclusions:**

In this comprehensive multicentre prospective-adjusted analysis of patients with SAH, hyponatraemia was investigated inconsistently and, for most patients, was not associated with changes in management or clinical outcome. This work establishes a basis for the development of evidence-based SAH-specific guidance for targeted screening, investigation and management of high-risk patients to minimise the impact of hyponatraemia on admission duration and to improve consistency of patient care.

**WHAT IS ALREADY KNOWN ON THIS TOPIC**

- Patients with subarachnoid haemorrhage (SAH) commonly develop hyponatraemia. However, a recent systematic review found no large prospective study of the association between hyponatraemia and clinical outcome which adjusted for potentially confounding variables. Findings from studies of general medical populations with symptomatic hyponatraemia may not apply to patients with SAH. Retrospective univariable analyses of patients with SAH are subjected to selection bias and confounding effects. In this context, a large prospective multicentre study assessing the management and outcome of hyponatraemia after SAH may inform the development of clinical guidance.

**WHAT THIS STUDY ADDS**

- Using adjusted, prespecified analyses of prospectively collected data from 407 patients with SAH, we found that although practice in the UK and Ireland generally did not adhere to European guidance for the screening, investigation and management of hyponatraemia after SAH, hyponatraemia was not associated with worse functional outcome. Furthermore, we identified risk factors for early and late hyponatraemia after SAH.

**HOW THIS STUDY MIGHT AFFECT RESEARCH, PRACTICE AND/OR POLICY**

- Intensive screening to identify hyponatraemia where this is unlikely to influence management decisions or clinical outcome is of limited value. This study establishes a basis for developing specific guidance for the focused screening, investigation and management of hyponatraemia after SAH

## Introduction

Subarachnoid haemorrhage (SAH) has an incidence of 6.9–9.0 per 100 000 person-years and a case fatality of 18%–44%.^1 2^ Hyponatraemia affects 27–44% of patients after SAH, most commonly from the syndrome of inappropriate antidiuretic hormone (SIADH), or cerebral salt wasting (CSW) syndrome.^3–5^ Hyponatraemia may be exacerbated by fluid administration for the treatment or prophylaxis of delayed cerebral ischaemia.^6^

Hyponatraemia after SAH has been inconsistently associated with seizures, neurological deficit, longer length of hospital stay and increased mortality.^3^ Interpretation of these associations is affected by variable timing of sodium measurement, potential selection biases and small sample sizes. There is no strong evidence to support common treatments for hyponatraemia. As such, the clinical significance of hyponatraemia after SAH is debated.^4 7 8^

## Aims

To investigate the clinical significance and treatment of hyponatraemia after SAH, we assessed the screening, investigation and management of hyponatraemia after SAH across UK neurosurgical units (NSUs). We aimed first to establish whether current practices reflected European Stroke Organisation (ESO) or European Society of Endocrinology (ESE) recommendations, second, to identify patient factors at admission, which are associated with hyponatraemia after SAH, and finally to determine whether hyponatraemia and its management were associated with short-term outcomes.

## Methods

### Design

Prospective multicentre audit of patients with spontaneous SAH admitted to participating NSUs in the UK and Ireland. The study protocol was prospectively uploaded to the British Neurosurgical Trainees Research Collaborative website: www.bntrc.org.uk/sash.

### Setting

We invited all 32 adult NSUs to participate. Collaborators identified eligible patients consecutively admitted within a 2-month period between October 2019 and March 2020. An interim review of recruitment extended permitted recruitment up to 4 months. We used a secure electronic system to collect anonymised data.^9^ The steering committee provided training to collaborators via interactive online seminars, written training materials and email. Within each unit, a neurosurgical registrar or consultant supervised case ascertainment and data collection by medical students. Data were reviewed and validated by the supervisor.

### Participants

Eligible patients were aged ≥18 years with spontaneous SAH diagnosed radiologically or by lumbar puncture. We excluded patients with traumatic SAH or lacking a prespecified minimum data set, including NSU admission duration and results of serum sodium measurements. Follow-up period ended on death, discharge from NSU or day 21 of NSU admission, whichever occurred earliest. For secondary analyses, only patients admitted to an NSU within 7 days of symptom onset were included.

### Variables and audit standards

Hyponatraemic periods were defined from measurement of serum sodium <135 mmol/L until a subsequent serum sodium measurement of ≥135 mmol/L was recorded.^10^

We defined audit standards using ESO^11^ and ESE^10^ guidance (table 1). We used the best postresuscitation findings recorded within 48 hours of admission to define Glasgow Coma Score and World Federation of Neurosurgical Societies (WFNS) grade.^12^ WFNS grade was dichotomised as poor (IV–V) or good grade (I–III).^13^ A neurosurgical registrar or consultant determined the modified Fisher score on diagnostic CT brain. This was subsequently dichotomised as those with thin or no visible SAH (grade 0–1) or those with thick SAH or intraventricular haemorrhage (grade 2–4).^14^ Neurosurgeons and neuroradiologists determined the probable causative lesion. Aneurysms were classified as anterior circulation if they occurred at, or anterior to, the posterior communicating artery. Lesion management was trichotomised as conservative, endovascular or open surgical.

**Table 1:** Audit standards derived from ESO and ESE guidelines

Standard	SAH population	Criteria
Standard 1	All patients	Serum sodium should be measured at least once every 48 hours
Standard 2	Hyponatraemia	Serum sodium should be measured at least once every 48 hours
Standard 3	Hyponatraemia	Volume status should be assessed daily while hyponatraemic
Standard 4	Hyponatraemia	Blood glucose, urinary sodium, urinary osmolality, serum osmolality and morning serum cortisol should be measured at least once while hyponatraemic

ESO, European Stroke Organisation; ESO, European Stroke Organisation; SAH, subarachnoid haemorrhage.

We categorised preadmission use of medications associated with risk of hyponatraemia as none, one or more than one.^15^ These included antihypertensives, antipsychotics, antidepressants, antiepileptic medications and proton pump inhibitors.^16–20^ We recorded pre-existing diagnoses of diabetes mellitus, chronic kidney disease, polycystic kidney disease, heart failure, adrenal insufficiency, hypothyroidism or hyponatraemia. Diagnoses of SIADH, CSW or other causes of hyponatraemia were recorded as determined by the treating team.

For each day of admission, volume status assessment was categorised as a bedside clinical assessment alone, or as fluid balance. Fluid balance was categorised as: positive (>500 mL), neutral (0–500 mL, to account for insensible losses) or negative (<0 mL). Target intake was similarly classified as high (target intake >3 L or >500 mL balance), neutral (intake 2–3 L or 0–500 mL balance), low (target intake <2 L or balance <0 mL). We included all measurements of serum sodium. We collected serum cortisol measurements if sampled prior to 09:00. Modified Rankin Scale (mRS) was determined at discharge and dichotomised for our primary outcome as slight or no dependency (0–2) versus dependency or death (3–6).^21^

### Statistical analysis

We performed data preparation and analysis using RStudio (V.1.3.1093) running R Core (V.3.6.1) and the following packages: survival (V.3.2–11), cmprsk (V.2.2–10), survminer (V.0.4.9), coxme (V.2.2–16), lme4 (V.1.1–27) and stats (V.3.6.1). We did not perform univariable analyses of patient characteristics to avoid multiple testing, in accordance with STROBE guidelines.^22^ For secondary analyses, the inception point was SAH symptom onset.

We investigated potential risk factors for first-ever hyponatraemia by complete case analyses using multivariable Cox regression models. We identified *a priori* set of putative variables associated with hyponatraemia. These were age, sex, previous diagnoses of conditions associated with hyponatraemia (as described), estimated glomerular filtration rate (eGFR) prior to SAH onset, WFNS grade, modified Fisher grade, aneurysm location and medications associated with hyponatraemia (as above).^3 23 24^ Fewer than 20 patients had one or more of the conditions associated with hyponatraemia, and eGFR was unknown for 328 patients. These two variables were removed from the set of covariates. For each covariate, we tested proportionality of hazards using Pearson product-moment correlations of Schoenfeld residuals with time. Patients who have died are unable to develop hyponatraemia and, therefore, censoring due to the competing risk of death was a potential source of bias. To address this, we performed a sensitivity analysis of competing risks using Fine and Gray proportional subdistribution hazards models of the same covariates as our primary analysis and plotted graphs of cumulative incidences for hyponatraemia and death. Furthermore, to address the potential for varying influences according to the NSU, each patient was managed in, we performed an exploratory mixed effects Cox regression using the same covariates as fixed effects with a random intercept of NSU.

We defined early and late hyponatraemia during our exploratory data analysis. Inspection of the cumulative incidence curves of hyponatraemia indicated the rate of hyponatraemia declined 10 days after SAH onset. We developed a Cox model of early hyponatraemia by restricting follow-up to 10 days after SAH onset. In a separate Cox model, late hyponatraemia was defined as a new episode of hyponatraemia occurring, or recurring 10 days or later after symptom onset, with model entry at 10 days.

To analyse associations of hyponatraemia with outcome, we used multiple logistic regression fit by maximum likelihood estimation, adjusting for prespecified covariates. These were selected during consensus meetings of the study steering committee as those demonstrated to have potential associations with SAH outcome.^25–27^ The primary outcome was dependency or death (mRS 3–6). The prespecified covariables were age, sex, modified Fisher grade, WFNS grade, aneurysm location and whether complications including vasospasm, ventriculitis or hydrocephalus occurred during admission up to 21 days, discharge or death. To account for residual confounding due to variable length of admission, we included duration of admission in days until 21 days, discharge or death as a covariate. A secondary analysis of dichotomised admission duration (fewer than 10 days vs greater than 10 days) was performed using the same covariates except for duration of admission. This time point was informed by the findings of our cumulative incidence analysis, which indicated that hyponatraemia incidence was maximal within 10 days of SAH onset. Because admission duration skewed by study closure at 21 days, linear regression was not appropriate. We performed exploratory subgroup analyses of patients with hyponatraemia. In this group, we examined possible relationships between hyponatraemia treatment, with sodium supplementation (with oral or intravenous) or administration of a negative or neutral fluid balance. Because of the relatively low frequency of death or dependency in these subgroups, adjusted analyses were not appropriate. We, therefore, present univariable data for subgroup analysis of outcome only, without inferential statistics. To consider potential random effects of NSU on the influence of hyponatraemia on outcome, we performed exploratory mixed effects analysis.

For all regression analyses, we carefully scrutinised the correlations and contingencies of linear and categorical covariates, respectively, to ensure that collinearity and separation did not occur.

### Regulatory approval

The audit protocol was approved by the audit, clinical and information governance committees of participating centres. Anonymised data were extracted from medical records into the audit database.

## Results

### Patient characteristics

**Figure 1:**
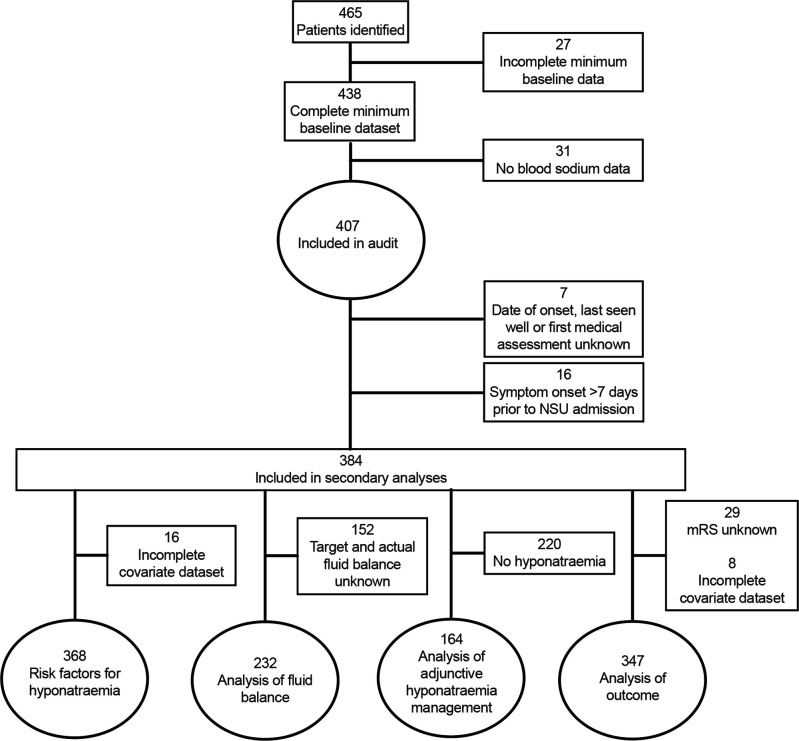
Flow diagram of patient inclusion and analysis. mRS, modified Rankin Scale; NSU, neurosurgical unit.

Seventy-five per cent (n/N=24/32) invited NSUs participated in this study (online supplemental table 1). Twenty-one per cent (n/N=5/24) were unable to complete a full period of case ascertainment prior to the early study closure at onset of the COVID-19 pandemic in March 2020. Of eight non-participating units, two declined participation and six were unable to commence case ascertainment prior to study closure. Of 465 potentially eligible patients, 407 eligible patients with spontaneous SAH were included (figure 1). Ineligible patients had similar clinical characteristics to eligible patients (online supplemental table 2). At least one measurement of serum sodium was recorded on 65% (n/N=3910/5452) follow-up days, with a total of 5976 measurements recorded. Fourty-three per cent (n/N=175/407) patients developed hyponatraemia, and 13% (n/N=54/407) patients had at least one measurement of serum sodium <130 mmol/L. 12% (n/N=48/407) patients died during the study period, of which 12 developed hyponatraemia prior to death. A diagnosis of SIADH or CSW as the cause of hyponatraemia was documented in 5% (n/N=9/175) and 3% (n/N=6/175) hyponatraemic patients, respectively. Patient characteristics are described in table 2 and online supplemental table 3).^28^

10.1136/svn-2022-001583.supp1 Supplementary data

**Table 2:** Clinical characteristics of 407 patients with spontaneous SAH admitted to a neurosurgical unit

Characteristic N (%)	Overall	Hyponatraemia	No hyponatraemia
Number of patients	407	175	232
Age at diagnosis in years: median (IQR)	58 (48–66)	60 (51–68)	56 (46–65)
Female sex	250 (61%)	114 (65%)	136 (59%)
Weight in kg: median (IQR)	73 (64–85)	71 (61–80)	76 (65–87)
Unknown	75 (18%)	27 (15%)	48 (21%)
Previous SAH	34 (8%)	17 (10%)	17 (7%)
Unknown	4 (0.98%)	0 (0%)	4 (1.7%)
Previous hyponatraemia	1 (0.3%)	1 (0.6%)	0 (0%)
Unknown	4 (0.98%)	0 (0%)	4 (1.7%)
Hyponatraemia-inducing drugs on admission			
None	247 (61%)	104 (59%)	143 (62%)
One	86 (21%)	40 (23%)	46 (20%)
Multiple	73 (18%)	31 (18%)	42 (18%)
Unknown	1 (0.3%)	0 (0%)	1 (0.4%)
WFNS grade			
I	232 (57%)	94 (54%)	138 (59%)
II	57 (14%)	38 (22%)	19 (8%)
III	25 (6%)	11 (6%)	14 (6%)
IV	47 (12%)	17 (10%)	30 (13%)
V	42 (20%)	13 (7%)	29 (13%)
Unknown	4 (0.98%)	2 (1.1%)	2 (0.9%)
Modified Fisher score			
0	13 (3%)	2 (1.1%)	11 (5%)
1	77 (19%)	27 (15%)	50 (22%)
2	56 (14%)	19 (11%)	37 (16%)
3	107 (26%)	55 (31%)	52 (22%)
4	139 (34%)	67 (38%)	72 (31%)
Unknown	15 (4%)	5 (3%)	10 (4%)
Aneurysm location			
Non-aneurysmal	104 (26%)	27 (15%)	77 (33%)
Anterior circulation	260 (64%)	120 (69%)	140 (60%)
Posterior circulation	43 (11%)	28 (16%)	15 (7%)
Treatment for vascular lesion			
Conservative	74 (18%)	17 (10%)	57 (25%)
Open surgical	48 (12%)	21 (12%)	27 (12%)
Endovascular	253 (62%)	129 (74%)	124 (53%)
Unknown	32 (8%)	8 (5%)	24 (10%)

SAH, subarachnoid haemorrhage; WFNS, World Federation of Neurosurgical Societies.

### Audit standards

We audited screening and monitoring of hyponatraemia (table 1). Considering each 48-hour interval from admission, or last serum sodium measurement, as a separate period, serum sodium was measured on a median 83% (IQR 64%–100%) of periods for each patient. Of 175 patients who developed hyponatraemia, serum sodium was measured on a median of 75% (IQR 50%–100%) of the 48-hour periods. Volume status assessment and measurement of serum and urine osmolalities, urine sodium, blood glucose and morning cortisol could be audited for 95% (n/N=166/175) patients with hyponatraemia. For these patients, volume status was documented on a median of 32% of days while hyponatraemic. The following were measured at least once following diagnosis of hyponatraemia: serum osmolality (n/N=49/166; 30%), urine osmolality (n/N=38/166; 23%), urine sodium (n/N=35/166; 21%), blood glucose (n/N=46/166; 28%), morning cortisol (n/N=7/166; 4%). Urine osmolality, serum osmolality and urinary sodium were measured together in 18% (n/N=30/166).

### Risk factors for hyponatraemia after SAH

**Figure 2:**
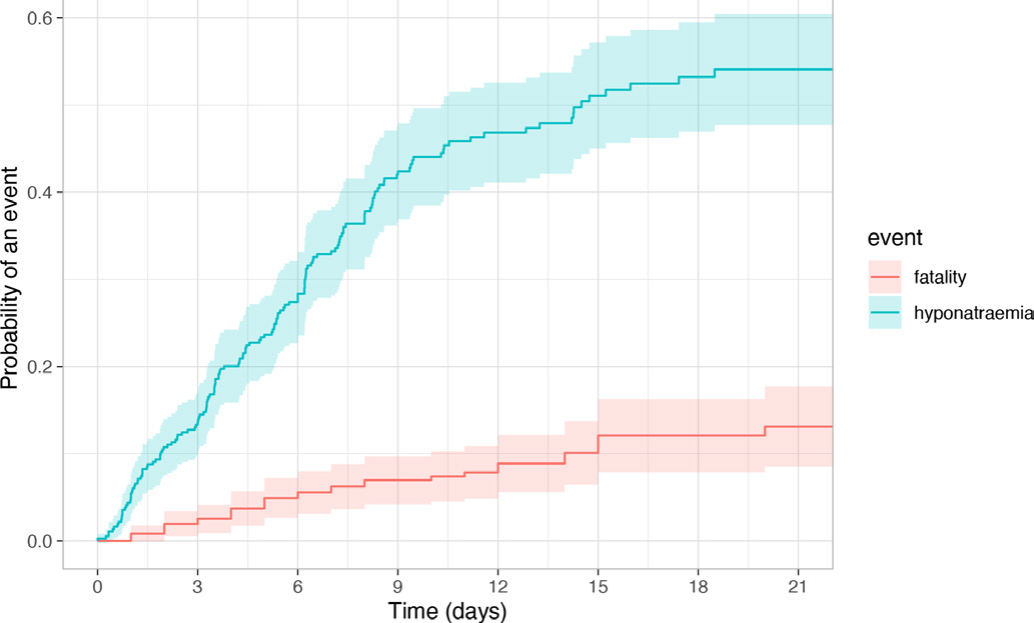
Cumulative incidence curves of cumulative hyponatraemia and cumulative fatality. Derived from cumulative incidence function of competing risk analysis. Shaded area represents 95% CI.

There were 368 patients admitted within 1 week of SAH onset who had complete data for analysis (figure 1). These had similar characteristics to the overall cohort (online supplemental table 2).^28^ Most first-ever episodes of hyponatraemia occurred within 10 days of SAH onset (89%; n/N=142/159; figure 2). 24% (n/N=53/218) patients admitted ≥10 days developed recurrent or denovo late hyponatraemia.

Risk of hyponatraemia was higher in patients with a modified Fisher grade 2–4 (adjusted HR (aHR) 1.7 (95% CI 1.1 to 2.7); p=0.022) and posterior circulation aneurysms (aHR 2.1 (CI 1.2 to 3.7); p=0.012) but was lower in those with WFNS grade IV–V (aHR 0.50 (CI 0.40 to 0.80) p=0.004) (table 3). Two sensitivity analyses accounting for the competing risk of death and random effects of NSU showed the same directions of adjusted association as our multivariable Cox regression, supporting our primary analysis (online supplemental tables 4 and 5).^28^

**Table 3:** Cox regression analysis of 368 patients with hyponatraemia after SAH

Covariable	aHR (95% CI)	P value
Age (years)	1.0 (1.0 to 1.0)	0.055
Male	1.2 (0.8 to 1.6)	0.35
WFNS grade IV–V	0.5 (0.4 to 0.8)	0.004
Fisher grade 2–4	1.7 (1.1 to 2.7)	0.022
Medications associated with hyponatraemia		
None	Reference	
One	1.0 (0.7 to 1.5)	0.91
Multiple	0.75 (0.5 to 1.2)	0.21
Aneurysm location		
Non-aneurysmal SAH	Reference	
Anterior circulation	1.3 (0.8 to 2.1)	0.26
Posterior circulation	2.1 (1.2 to 3.7)	0.012

AhR, adjusted hyponatraemia-specific; SAH, subarachnoid haemorrhage; WFNS, World Federation of Neurosurgical Societies.

We hypothesised that hyponatraemia occurring denovo, or recurring, at 10 or more days after SAH may have a distinct risk profile to early hyponatraemia. We, therefore, stratified our data set to identify risk factors for late hyponatraemia occurring de novo or recurring from day 10 onwards. Cox regression demonstrated associations between increased age (aHR 1.03 (CI 1.00 to 1.05); p=0.027) and previous early hyponatraemia (aHR 2.7 (CI 1.5 to 4.9); p=0.001), with late hyponatraemia; online supplemental tables 2 and 6),^28^ following adjustment for the same factors as described previously. Analysis of early hyponatraemia occurring before day 10 showed similar associations to our unstratified analysis (online supplemental table 7).^28^

### Management

A target intake or actual daily fluid balance was documented for 232 patients (figure 1). Target fluid intake was available for 195 patients, of which 29% (n/N=56/195) had documented during both hyponatraemic and non-hyponatraemic periods. 125 patients had actual daily fluid balance documented, of which it was documented during both hyponatraemic and non-hyponatraemic periods for 50% (n/N=62/125). The percentage of time for which a fluid daily intake over 3 L was targeted or a positive fluid balance was actually achieved did not differ between periods when patients were hyponatraemic or not hyponatraemic. The median percentage of days targeting >3 L intake during hyponatraemic periods was 100% (IQR 0–100) and 100% (IQR 12–100) during non-hyponatraemic periods. The median percentage of days for which a positive fluid balance occurred during hyponatraemic periods was 50% (IQR 0%–75%) and 50% (IQR 33%–95%) during non-hyponatraemic periods.

Several patients who developed hyponatraemia received adjunctive treatments (figure 1): 26% (n/N=42/164) patients received supplementary sodium (oral sodium 15; hypertonic saline 33 patients), 1.2% (n/N=2/164) received a diuretic and none received tolvaptan, urea or demeclocycline.

### Outcome

End of follow-up mRS was recorded for 92% (n/N=355/384) patients (figure 1), of whom 35% (n/N=133/384) were dead or dependent at discharge or 21 days (online supplemental table 8).^28^ In a logistic regression model with 347 patients (online supplemental table 2)^28^ with mRS and covariable data available, hyponatraemia was associated with lower odds of death or dependency (table 4, online supplemental table 9),^28^ following adjustment for other predictors of outcome after SAH and admission duration. The direction of this association was not influenced by the exploratory addition of a random intercept representing NSU to the model (online supplemental table 10). Hyponatraemia was associated with longer admission duration following adjustment other predictors of outcome after SAH (table 4) in a model of 355 patients (online supplemental table 2)^28^ with complete data. Exploratory mixed effects analysis of length of stay with inclusion of a random intercept of NSU was not possible as resulted in perfect separation.

**Table 4:** Multiple logistic regression analyses of risks of death or dependency and of neurosurgical admission of 10 days or more after SAH

Outcome	Covariable	aOR (95% CI)	P value
**Death or dependency (mRS 3–6): N=347**			
	Hyponatraemia	0.35 (0.17 to 0.69)	0.003
	Age (years)	1.1 (1.0 to 1.1)	<0.001
	Male	1.1 (0.55 to 2.3)	0.76
	WFNS grade IV–V	33 (14 to 97)	<0.001
	Fisher grade 2–4	5.0 (1.8 to 16)	0.003
	Aneurysm location		
	Non-aneurysmal SAH	Reference	
	Anterior circulation	3.2 (1.2 to 8.7)	0.019
	Posterior circulation	3.5 (1.0 to 13)	0.050
	Complications	2.9 (1.5 to 6.0)	0.003
	Admission duration (days)	1.0 (0.97 to 1.1)	0.38
**Neurosurgical admission of 10 days or more: N=355**			
	Hyponatraemia	3.2 (1.8 to 5.7)	<0.001
	Age (years)	1.0 (0.99 to 1.0)	0.41
	Male	1.1 (0.64 to 2.0)	0.71
	WFNS grade IV–V	0.61 (0.30 to 1.21)	0.15
	Fisher grade 2–4	(1.73 (0.91 to 3.3)	0.09
	Aneurysm location		
	Non-aneurysmal SAH	Reference	
	Anterior circulation	5.4 (2.9 to 10.2)	<0.001
	Posterior circulation	7.7 (2.6 to 26.5)	<0.001
	Complications	2.7 (1.5 to 5.0)	<0.001

Complications include a diagnosis of vasospasm, ventriculitis or hydrocephalus.

aOR, adjusted OR; mRS, modified Rankin Scale; SAH, subarachnoid haemorrhage; WFNS, World Federation of Neurosurgical Societies.

In the subgroup of 43% (n/N=152/355) patients who developed hyponatraemia during admission, the percentage of hyponatraemic period where a negative or neutral fluid balance was achieved was similar for both outcome groups at end of follow-up (mRS 3–6 vs mRS 0–2: median 50 (IQR 18–98) vs 50 (IQR 33–100); online supplemental table 11).^28^ Likewise, use of oral or intravenous sodium supplementation was similar for both outcome groups (mRS 3–6 vs mRS 0–2: n/N=13/51 (25%) vs n/N=27/101 (27%)). Patients treated with supplementary sodium while hyponatraemic had a median hyponatraemic period of 8 days (IQR 5–12) compared with 4 days (IQR 2–7; online supplemental figure 1A)^28^ for those who were not. We observed no evidence of a relationship between achievement of a low/neutral fluid balance and hyponatraemia duration, including in the subgroup of patients with moderate–severe hyponatraemia (online supplemental figure 1B, C).^28^

## Discussion

In this prospective, multicentre, study of patients with SAH, we found that guidance for the screening and investigation of hyponatraemia was adhered to infrequently.^10 11^ This contrasts findings of a linked survey in which all NSUs subjectively reported measurement of serum sodium daily or on alternate days, and the majority reported the routine paired measurement of serum and urine osmolalities as well as urinary sodium to investigate hyponatraemia:^7^ in practice, they were measured in just 18% of cases.

Clinical teams managing patients with SAH do not prioritise monitoring of serum sodium or distinguishing between potential causes of hyponatraemia. Without a diagnosis, aetiology-based management guidance is challenging to apply.^10^ However, there was no evidence that restrictive or normovolaemic fluid strategies recommended by ESO were more frequently targeted or achieved during periods of hyponatraemia compared with non-hyponatraemic periods.^11^ This may reflect concern that normovolaemic or restrictive fluid management strategies could precipitate or exacerbate cerebral hypoperfusion or arterial vasospasm.^29 30^ Where restrictive or normovolaemic fluid management strategies were achieved, no relationship between their employment and duration of hyponatraemia was observed. Although most instances of hyponatraemia after SAH are attributable to SIADH, other causes such as CSW, diuretic medication use and vomiting are possible and may not respond to fluid restriction.^5 10^

Hyponatraemia had an adjusted association with lower levels of death and dependency. This may reflect the management of patients with hyponatraemia in a higher level of care having lower risks of hypernatraemia. If patients with hyponatraemia are managed at a higher level of care, this might modify risk of poor outcome. This phenomenon has been previously observed in an unadjusted analysis.^8^ In a systematic review, some studies found hyponatraemia to be associated with poor outcomes, but these associations were absent in larger, adjusted analyses.^3 31–33^ Such uncertainty could be addressed through a study design using standardised sampling protocol. However, recruitment of patients with severe SAH to such a study which would require obtaining informed consent might be challenging and drive a selection bias. Although we identified associations between hyponatraemia and early clinical outcomes, we did not evaluate intermediate or longer term outcomes. It remains possible that hyponatraemia may influence more subtle measures of outcome or function, including cognition and return to work. As these are outcomes which valued highly by patients, these merit further analysed in a dedicated prospective observational study. As most of our cohort did not have moderate or severe hyponatraemia (<130 mmol/L), our results may not be generalisable to these patients.

Existing guidance for screening, investigation and management of hyponatraemia after SAH is not applied and a new approach is required. Invasive screening by frequent blood testing and prolonged inpatient stays which are not associated with changes to patient management have economic and welfare costs. Our data suggest that targeted sodium screening up to 10 days after SAH onset is most likely to identify abnormality. Beyond 10 days, few patients develop hyponatraemia. Older age and hyponatraemia in the preceding 10 days were associated with late hyponatraemia and these could be prioritised for screening for late hyponatraemia and its detection should be likely to trigger intervention.

During the early period, patients with posterior circulation aneurysms and greater Fisher scores could be prioritised for frequent sodium monitoring. The association of poor WFNS score with reduced risk of hyponatraemia may relate to critical care or expectant management of severely unwell patients and consequent differences in blood sampling frequency. Associations of aneurysm territory with hyponatraemia have been inconsistently reported and ours is the first adjusted analysis to identify an association between aneurysm location with hyponatraemia.^3^ The association of posterior circulation aneurysms with hyponatraemia may reflect altered posterior circulation arterial supply to middle and posterior hypothalamus.^34^ There is no strong association between posterior circulation aneurysms and aneurysm size compared, so this seems unlikely to be a confounding factor.^35^

Further research is needed to identify which, if any, patients can benefit from targeted therapy to correct hyponatraemia after SAH, and indeed what treatments are effective. External validation of our findings in similarly well-designed, adjusted analyses is warranted. Randomised studies of sodium supplementation or fluid balance manipuplation for hyponatraemia after SAH are lacking and historic trials of fluid management strategies in SAH have struggled to recruit.^29 36^ Unless the challenges of conducting such trials in SAH populations can be overcome, consensus-based guidance, informed by observational studies, may be required to rationalise the management of hyponatraemia in patients with SAH. We, therefore, propose undertaking of a Delphi process involving patients and relevant specialty experts.^37^

Our study has some strengths. As a prospective, multicentre, hospital-based study of consecutive patients with SAH in the UK and Ireland, the potential for selection bias is minimised and our findings are widely generalisable. This is supported by our exploratory mixed effects analyses, which found no evidence of an effect of NSU on any outcome. We collected data on all measurements of serum sodium and so our definition of hyponatraemic and non-hyponatraemic periods is robust. Although our follow-up period was relatively short, functional deficits attributable to acute or subacute complications of SAH are likely to be evident by 21 days and predict longer term outcomes.^38 39^ There are some weaknesses. Case acquisition was halted early due to the COVID-19 pandemic and so certain centres were unable to contribute full data. Patients who are managed out with an NSU may have different characteristics to those in our study.^38^ We used data recorded in the routine management of patients and therefore clinical assessments which were performed, but not documented, were omitted. By using results of blood tests extracted from patient laboratory records rather than a standardised scheduled blood sampling schedule, it is possible that some patients with minimally symptomatic hyponatraemia were not diagnosed. Conversely, it is also possible that hyponatraemia might be more frequently detected in patients with severe illness or critical care admission who undergo frequent blood sampling. We did not account for variation in pre-SAH dependency, which could be associated with hyponatraemia. These potential information biases would be expected to reduce the observed association between hyponatraemia and better outcomes and, therefore we do not think they are likely to strongly influence our conclusions.

## Conclusions

Hyponatraemia after SAH is inconsistently investigated and managed. There is an urgent need for pragmatic, consensus-based guidelines. Hyponatraemia is uncommon beyond 10 days after SAH, but patients with older age and previous early hyponatraemia have higher risk. Admission to NSUs after SAH may not need to be prolonged because of hyponatraemia alone.

## Data Availability

Data are available upon reasonable request. Data produced in this study may be available to reasonable requests with appropriate institutional agreement. Requests for data should be made to the corresponding author.
