# Supplementary material for: Prospective, multicentre study of screening, investigation and management of hyponatraemia after subarachnoid haemorrhage in the UK and Ireland
Source: Stroke Vasc Neurol. 2022 Sep 23;8(3):207–16. doi: 10.1136/svn-2022-001583 (PMC10359796; doi:10.1136/svn-2022-001583)

A prospective, multicentre study of the investigation and management of hyponatraemia after subarachnoid haemorrhage: Supplementary Material

Contents

Supplementary table 1: Participating neurosurgical units. 2
Supplementary table 2: Patient characteristics per analysis....3
Supplementary table 3: Previous medical history and medications prior to admission in 407 patients with spontaneous SAH admitted to a neurosurgical unit. 7
Supplementary table 4: Fine and Grey competing risk regression analysis of hyponatraemia and fatality after SAH 8
Supplementary table 5: Mixed effect Cox regression analysis of hyponatraemia after SAH....9
Supplementary table 6: Cox regression analysis of late hyponatraemia after SAH...10
Supplementary table 7: Cox regression analysis of early hyponatraemia after SAH.11
Supplementary table 8: Outcomes of 384 patients with spontaneous SAH admitted to a neurosurgical unit. 12
Supplementary table 9: Factors associated with death or dependency (mRS 3-6) for 355 patients with spontaneous SAH admitted to a neurosurgical unit. 13
Supplementary table 10: Mixed effects multiple logistic regression analyses of risks of death or dependency in 347 patients after SAH. 14
Supplementary table 11: Factors associated with death or dependency (mRS 3-6) for 152 patients with hyponatraemia after spontaneous SAH admitted to a neurosurgical unit. 15
Supplementary figure 1: Exploratory analyses treatment and duration of hyponatraemia. 16

Loan JJM, *et al. Stroke Vasc Neurol* 2023; 8:e001583. doi: 10.1136/svn-2022-001583

Supplementary table 1: Participating neurosurgical units.
2002 estimates adjusted for UK population growth. Note that total population figures are not accurate due to overlapping catchment areas. Farling PA. The neuroanaesthesia workforce in Great Britain and Ireland. Anaesthesia. 2003 Jan;58(1):17–23.

Neurosurgical unit (city)	Country	Catchment population (millions)
Addenbrooke's Hospital (Cambridge)	England	3.2
Beaumont Hospital (Dublin)	Republic of Ireland	3.6
Bristol Institute of Clinical Neuroscience (Bristol)	England	2.9
Cork University Hospital (Cork)	Republic of Ireland	1.3
Derriford Hospital (Plymouth)	England	1.9
Hull Royal Infirmary (Hull)	England	1.7
James Cook University Hospital (Middlesborough)	England	1.3
John Radcliffe Hospital (Oxford)	England	3.9
King's College Hospital (London)	England	3.9
Leeds General Infirmary (Leeds)	England	3.9
National Hospital for Neurology and Neurosurgery (London)	England	3.0
Ninewells Hospital (Dundee)	Scotland	0.8
Nottingham University Hospital (Nottingham)	England	3.9
Princess Royal Hospital (Brighton)	England	1.6
Queen Elizabeth University Hospital (Glasgow)	Scotland	3.6
Queen's Hospital (London)	England	2.6
NHS Lothian Department of Clinical Neurosciences (Edinburgh)	Scotland	2.1
Royal Preston Hospital (Preston)	England	2.3
Royal Stoke University Hospital (Stoke)	England	1.7
Royal Victoria Infirmary (Newcastle)	England	3.9
St Bartholomew's Hospital (London)	England	2.2
University Hospital of Wales (Cardiff)	Wales	2.0
University Hospital Southampton (Southampton)	England	3.6
The Walton Centre NHS Foundation trust (Liverpool)	England	3.9

Loan JJM, *et al*. *Stroke Vasc Neurol* 2023; 8:e001583. doi: 10.1136/svn-2022-001583

Supplementary table 2: Patient characteristics per analysis

IQR interquartile range; d=day

**Analysis**	**Descriptive**		**Risk factors for hyponatraemia**		**Management**	**Risk factors for death/dependency**	**Risk factors for >10d admission**
**Group/subgroup**	**All eligible patients**	**Missing minimum baseline or sodium data**	**All with complete data**	**≥10 day admission**	**Fluid balance target or achieved recorded**	**All with complete data**	**All with complete data**
**Characteristic, n(%)**							
Number of patients	407	58	368	218	232	347	355
Hyponatraemia during analysis period	175 (43%)	NA	159 (43%)	53 (24%)	139 (60%)	148 (43%)	152 (43%)
Age at diagnosis in years: median (IQR)	58 (48-66)	60 (53-68)	58 (47-66)	59 (50-67)	59 (51-66)	57 (47-65)	57 (47-65)
Unknown	0 (0%)	9 (16%)	0 (0%)	0 (0%)	0 (0%)	0 (0%)	0 (0%)
Female sex	250 (61%)	30 (50%)	232 (63%)	145 (67%)	154 (66%)	216 (62%)	222 (63%)
Unknown	0 (0%)	8 (14%)	0 (0%)	0 (0%)	0 (0%)	0 (0%)	0 (0%)
Weight in kg: median (IQR)	73 (64-85)	64 (46-74)	73 (63-85)	72 (60-82)	72 (62-83)	73 (63-85)	73 (63-85)
Unknown	75 (18%)	14 (24%)	62 (17%)	27 (12%)	17 (7%)	55 (16%)	56 (16%)
Previous SAH	34 (8%)	8 (14%)	31 (8%)	19 (9%)	18 (8%)	29 (8%)	29 (8%)
Unknown	4 (0.98%)	0 (0%)	4 (1%)	1 (0.5%)	0 (0%)	4 (1%)	4 (1%)
Previous hyponatraemia	1 (0.3%)	0 (0%)	0 (0%)	0 (0%)	0 (0%)	0 (0%)	0 (0%)
Unknown	4 (0.98%)	0 (0%)	4 (1%)	1 (0.5%)	0 (0%)	4 (1%)	4 (1%)

**Analysis**	**Descriptive**		**Risk factors for hyponatraemia**		**Management**	**Risk factors for death/dependency**	**Risk factors for >10d admission**
**Group/subgroup**	**All eligible patients**	**Missing minimum baseline or sodium data**	**All with complete data**	**≥10 day admission**	**Fluid balance target or achieved recorded**	**All with complete data**	**All with complete data**
**Characteristic, n(%)**							
Hyponatraemia-inducing drugs on admission							
None	247 (61%)	23 (40%)	227 (62%)	131 (60%)	138 (59%)	211 (61%)	217 (61%)
One	86 (21%)	21 (36%)	77 (21%)	46 (21%)	51 (22%)	73 (21%)	74 (21%)
Multiple	73 (18%)	13 (22%)	64 (17%)	41 (19%)	43 (19%)	62 (18%)	63 (18%)
Unknown	1 (0.3%)	1 (2%)	0 (0%)	0 (0%)	0 (0%)	1 (0.3%)	1 (0.3%)
WFNS grade							
I	232 (57%)	22 (38%)	209 (57%)	109 (50%)	132 (57%)	198 (57%)	202 (57%)
II	57 (14%)	8 (14%)	52 (14%)	40 (18%)	39 (17%)	49 (14%)	51 (14%)
III	25 (6%)	0 (0%)	25 (7%)	15 (7%)	13 (6%)	22 (6%)	22 (6%)
IV	47 (12%)	8 (14%)	43 (12%)	35 (16%)	28 (12%)	39 (11%)	40 (11%)
V	42 (20%)	3 (5%)	39 (11%)	19 (9%)	18 (8%)	39 (11%)	40 (11%)
Unknown	4 (0.98%)	17 (29%)	0 (0%)	0 (0%)	2 (0.86%)	0 (0%)	0 (0%)
Modified Fisher score							
0	13 (3%)	1 (2%)	13 (4%)	1 (0.5%)	6 (3%)	13 (4%)	13 (4%)
1	77 (19%)	13 (22%)	66 (18%)	33 (15%)	42 (18%)	66 (19%)	66 (19%)

**Analysis**	**Descriptive**		**Risk factors for hyponatraemia**		**Management**	**Risk factors for death/dependency**	**Risk factors for >10d admission**
**Group/subgroup**	**All eligible patients**	**Missing minimum baseline or sodium data**	**All with complete data**	**≥10 day admission**	**Fluid balance target or achieved recorded**	**All with complete data**	**All with complete data**
**Characteristic, n(%)**							
2	56 (14%)	7 (12%)	52 (14%)	27 (12%)	28 (12%)	50 (14%)	53 (15%)
3	107 (26%)	8 (14%)	103 (28%)	64 (29%)	70 (30%)	99 (29%)	101 (28%)
4	139 (34%)	13 (22%)	134 (36%)	93 (43%)	82 (35%)	119 (34%)	122 (34%)
Unknown	15 (4%)	16 (28%)	0 (0%)	0 (0%)	4 (2%)	0 (0%)	0 (0%)
Aneurysm location							
Non-aneurysmal	104 (26%)	15 (26%)	89 (24%)	25 (11%)	43 (19%)	83 (24%)	84 (24%)
Anterior circulation	260 (64%)	26 (44%)	239 (65%)	159 (73%)	158 (68%)	225 (65%)	232 (65%)
Posterior circulation	43 (11%)	4 (7%)	181 (52%)	34 (16%)	31 (13%)	39 (11%)	39 (11%)
Unknown	0 (0%)	13 (22%)	0 (0%)	0 (0%)	0 (0%)	0 (0%)	0 (0%)
Treatment for vascular lesion							
Conservative	74 (18%)	14 (24%)	67 (18%)	19 (9%)	31 (13%)	65 (19%)	67 (19%)
Open surgical	48 (12%)	6 (10%)	42 (11%)	33 (15%)	26 (11%)	40 (12%)	41 (12%)
Endovascular	253 (62%)	23 (40%)	233 (63%)	159 (73%)	162 (70%)	220 (63%)	225 (63%)

Analysis	Descriptive		Risk factors for hyponatraemia		Management	Risk factors for death/dependency	Risk factors for >10d admission
**Group/subgroup**	**All eligible patients**	**Missing minimum baseline or sodium data**	**All with complete data**	**≥10 day admission**	**Fluid balance target or achieved recorded**	**All with complete data**	**All with complete data**
**Characteristic, n(%)**							
Unknown	32 (8%)	15 (26%)	26 (7%)	7 (3%)	13 (6%)	22 (6%)	22 (6%)

Loan JJM, *et al. Stroke Vasc Neurol* 2023; 8:e001583. doi: 10.1136/svn-2022-001583

Supplementary table 3: Previous medical history and medications prior to admission in 407 patients with spontaneous SAH admitted to a neurosurgical unit. SD standard deviation; IQR interquartile range

Characteristic N (%)	Overall	Hyponatraemia	No hyponatraemia
Number of patients	407	175	232
**Past medical history**			
Diabetes mellitus	13 (3%)	7 (4%)	13 (6%)
Unknown	4 (0.98%)	0 (0%)	4 (1.7%)
Chronic kidney disease	9 (2%)	2 (1.1%)	7 (3%)
Unknown	4 (0.98%)	0 (0%)	4 (1.7%)
Polycystic kidney disease	3 (0.7%)	0 (0%)	3 (1.3%)
Unknown	4 (0.98%)	0 (0%)	4 (1.7%)
Cardiac failure	3 (0.7%)	1 (0.6%)	2 (0.9%)
Unknown	4 (0.98%)	0 (0%)	4 (2%)
Adrenal insufficiency	1 (0.25%)	1 (0.6%)	0 (0%)
Unknown	4 (0.98%)	0 (0%)	4 (2%)
Hypothyroidism	13 (3%)	8 (5%)	5 (2%)
Unknown	4 (0.98%)	0 (0%)	4 (2%)
**Hyponatraemia-inducing drugs on admission**			
Antihypertensive	97 (24%)	41 (23%)	56 (24%)
Unknown	1 (0.25%)	0 (0%)	1 (0.4%)
Antipsychotic	9 (2%)	6 (3.4%)	3 (1.3%)
Unknown	1 (0.25%)	0 (0%)	1 (0.4%)
Antidepressant	54 (13%)	28 (16%)	26 (11%)
Unknown	1 (0.25%)	0 (0%)	1 (0.4%)
Antiepileptic	9 (2%)	2 (1.1%)	7 (3%)
Unknown	1 (0.25%)	0 (0%)	1 (0.4%)
Proton pump inhibitor	42 (10%)	17 (10%)	25 (11%)
Unknown	1 (0.25%)	0 (0%)	1 (0.4%)

Supplementary table 4: Fine and Grey competing risk regression analysis of hyponatraemia and fatality after SAH

N=368 patients; aSHR: adjusted subdistribution hyponatraemia hazard ratio.

**Covariable**	**aSHR (95% confidence interval)**	**P value**
Age (years)	1.0(1.00-1.03)	0.043
Male	1.2(0.8-1.6)	0.37
WFNS grade IV-V	0.5(0.3-0.7)	<0.001
Fisher grade 2-4	1.6(1.1-2.5)	0.026
Medications associated with hyponatraemia		
None	Reference	
One	1.0(0.7-1.5)	0.92
Multiple	0.78(0.5-1.2)	0.26
Aneurysm location		
Non-aneurysmal SAH	Reference	
Anterior circulation	1.3(0.8-2.2)	0.24
Posterior circulation	2.2(1.3-3.8)	0.004

Loan JJM, *et al. Stroke Vasc Neurol* 2023; 8:e001583. doi: 10.1136/svn-2022-001583

Supplementary table 5: Mixed effect Cox regression analysis of hyponatraemia after SAH

N=368 patients; aHR: adjusted hyponatraemia hazard ratio.

**Fixed effects**	**aHR (95% confidence interval)**	**P value**
Age (years)	1.01(1.00-1.03)	0.047
Male	1.1(0.8-1.6)	0.40
WFNS grade IV-V	0.5(0.3-0.8)	0.004
Fisher grade 2-4	1.7(1.05-2.7)	0.031
Medications associated with hyponatraemia		
None	Reference	
One	1.1(0.7-1.6)	0.64
Multiple	0.75(0.5-1.2)	0.23
Aneurysm location		
Non-aneurysmal SAH	Reference	
Anterior circulation	1.2(0.8-2.0)	0.42
Posterior circulation	2.1(1.2-3.8)	0.014
**Random intercept**	**Standard deviation**	
Hospital	0.34	

Supplementary table 6: Cox regression analysis of late hyponatraemia after SAH. n/N=53/218 patients admitted ≥10 days developed late hyponatraemia; aHR: adjusted hyponatraemia-specific hazard ratio

.

**Covariable**	**aHR (95% confidence interval)**	**P value**
Early hyponatraemia after SAH	2.7(1.5-5.0)	0.001
Age (years)	1.0(1.0-1.1)	0.027
Male	1.0(0.5-1.8)	0.94
WFNS grade IV-V	0.90(0.46-1.7)	0.74
Fisher grade 2-4	0.87(0.38-2.00)	0.74
Medications associated with hyponatraemia		
None	Reference	
One	0.74(0.37-1.5)	0.39
Multiple	1.3(0.66-2.7)	0.44
Aneurysm location		
Non-aneurysmal SAH	Reference	
Anterior circulation	3.4(0.80-14.6)	0.10
Posterior circulation	2.5(0.5-11.9)	0.25

Supplementary table 7: Cox regression analysis of early hyponatraemia after SAH. N=368; aHR: adjusted hyponatraemia-specific hazard ratio

**Covariable**	**aHR (95% confidence interval)**	**P value**
Age (years)	1.0(1.0-1.0)	0.10
Male	1.3(0.88-1.8)	0.21
WFNS grade IV-V	0.54(0.34-0.85)	0.01
Fisher grade 2-4	1.8(1.1-2.8)	0.02
Medications associated with hyponatraemia		
None	Reference	
One	1.0(0.68-1.6)	0.88
Multiple	0.77(0.47-1.2)	0.28
Aneurysm location		
Non-aneurysmal SAH	Reference	
Anterior circulation	1.3 (0.80-2.1)	0.28
Posterior circulation	2.2 (1.2-4.00)	0.009

Loan JJM, *et al. Stroke Vasc Neurol* 2023; 8:e001583. doi: 10.1136/svn-2022-001583

Supplementary table 8: Outcomes of 384 patients with spontaneous SAH admitted to a neurosurgical unit.

IQR interquartile range

**Characteristic N (%)**	**Overall**	**Hyponatraemia**	**No hyponatraemia**
Number of patients	384	164	220
Death or dependency (mRS 3-6)	133 (35%)	51 (31%)	82 (37%)
Unknown	29 (8%)	12 (7%)	17 (8%)
mRS at 21 days or discharge			
0	85 (22%)	25 (15%)	60 (27%)
1	103 (27%)	58 (35%)	45 (20%)
2	34 (9%)	18 (11%)	16 (7%)
3	34 (9%)	17 (10%)	17 (8%)
4	30 (8%)	13 (8%)	17 (8%)
5	23 (6%)	9 (5%)	14 (6%)
6	46 (12%)	12 (7%)	34 (15%)
Unknown	29 (8%)	12 (7%)	17 (8%)
Duration of admission: median (days; IQR)	13 (8-22)	16 (11-22)	10 (6-20)
Conditions diagnosed during admission			
Cerebral arterial vasospasm	93 (24%)	48 (29%)	45 (20%)
Unknown	20 (5%)	8 (5%)	12 (5%)
Ventriculitis	20 (5%)	11 (7%)	9 (4%)
Unknown	21 (5%)	9 (5%)	12 (5%)
Hydrocephalus	128 (33%)	60 (37%)	68 (31%)
Unknown	19 (5%)	9 (5%)	12 (5%)
Discharged to same setting as at admission	220 (57%)	96 (59%)	124 (56%)
Unknown	21 (5%)	8 (5%)	13 (6%)

Loan JJM, *et al. Stroke Vasc Neurol* 2023; 8:e001583. doi: 10.1136/svn-2022-001583

Supplementary table 9: Factors associated with death or dependency (mRS 3-6) for 355 patients with spontaneous SAH admitted to a neurosurgical unit.
SD standard deviation; IQR interquartile range

Characteristic N (%)	Overall	Dead or dependent	Independent
Number of patients	355	133	222
Hyponatraemia	156	51 (38%)	101 (45%)
Duration of admission: median (days; IQR)	13 (8-22)	21 (2-22)	11 (8-16)
Admission duration >10 days	240 (68%)	99 (74%)	141 (64%)
WFNS grade			
I	203 (57%)	26 (20%)	177 (80%)
II	50 (14%)	22 (17%)	28 (13%)
III	22 (6%)	13 (10%)	9 (4%)
IV	39 (11%)	34 (26%)	5 (2%)
V	39 (11%)	37 (28%)	2 (0.9%)
Unknown	2 (0.6%)	1 (0.8%)	1 (0.5%)
Modified Fisher score			
0	13 (4%)	0 (0%)	13 (5.9%)
1	66 (19%)	5 (4%)	61 (27%)
2	50 (14%)	21 (16%)	29 (13%)
3	99 (28%)	26 (20%)	73 (33%)
4	119 (34%)	80 (60%)	39 (18%)
Unknown	8 (2%)	1 (0.8%)	7 (3%)
Aneurysm location			
Non-aneurysmal	87 (25%)	15 (11%)	72 (32%)
Anterior circulation	228 (64%)	100 (75%)	128 (58%)
Posterior circulation	40 (11%)	18 (14%)	22 (9.9%)
Complications during admission	165 (46%)	96 (72%)	69 (31%)
Cerebral arterial vasospasm	89 (25%)	53 (40%)	36 (16%)
Ventriculitis	20 (6%)	17 (13%)	3 (1%)
Hydrocephalus	124 (35%)	79 (59%)	45 (20%)
Discharged to same setting as at admission	216 (61%)	25 (19%)	191 (86%)

Supplementary table 10: Mixed effects multiple logistic regression analyses of risks of death or dependency in 347 patients after SAH.

Complications include a diagnosis of vasospasm, ventriculitis or hydrocephalus.
aOR: adjusted odds ratio

**Fixed effects**	**aOR (95% confidence interval)**	**P value**
Hyponatraemia	0.34 (0.17-0.69)	0.003
Age (years)	1.1 (1.0-1.1)	<0.001
Male	1.1 (0.55-2.3)	0.73
WFNS grade IV-V	35 (13-96)	<0.001
Fisher grade 2-4	5.0 (1.8-15)	0.003
Aneurysm location		
Non-aneurysmal SAH	Reference	
Anterior circulation	3.2 (1.2-8.8)	0.019
Posterior circulation	3.7 (1.0-14)	0.050
Complications	3.0 (1.5-6.1)	0.003
Admission duration (days)	1.0 (0.97-1.1)	0.36
**Random intercept**	**Standard deviation**	
Hospital	0.24	

Loan JJM, *et al. Stroke Vasc Neurol* 2023; 8:e001583. doi: 10.1136/svn-2022-001583

Supplementary table 11: Factors associated with death or dependency (mRS 3-6) for 152 patients with hyponatraemia after spontaneous SAH admitted to a neurosurgical unit.

SD standard deviation; IQR interquartile range

Characteristic N (%)	Overall	Dead or dependent	Independent
Number of patients	152	51	101
Hyponatraemia	152 (100%)	51 (100%)	101 (100%)
% Hyponatraemic period negative-neutral fluid balance was achieved: median (IQR)	50 (25-100)	50 (18-98)	50 (33-100)
Unknown	55 (35%)	17 (33%)	38 (38%)
Sodium supplementation prescribed during hyponatraemic period	40 (26%)	13 (25%)	27 (27%)
Duration of admission: median (days; IQR)	16 (11-22)	22 (16-22)	14 (10-19)
Admission duration >10 days	129 (85%)	46 (90%)	83 (82%)
WFNS grade			
I	82 (54%)	13 (25%)	69 (68%)
II	32 (21%)	10 (20%)	22 (22%)
III	9 (6%)	4 (8%)	5 (5%)
IV	15 (10%)	13 (25%)	2 (2%)
V	12 (8%)	10 (20%)	2 (2%)
Unknown	2 (1%)	1 (2%)	1 (1%)
Modified Fisher score			
0	2 (1%)	0 (0%)	2 (2%)
1	21 (14%)	1 (2%)	20 (20%)
2	16 (11%)	7 (14%)	9 (9%)
3	52 (34%)	10 (20%)	42 (42%)
4	57 (38%)	32 (63%)	25 (25%)
Unknown	4 (3%)	1 (2%)	3 (3%)
Aneurysm location			
Non-aneurysmal	22 (14%)	1 (2%)	21 (21%)
Anterior circulation	102 (67%)	39 (76%)	63 (62%)
Posterior circulation	28 (18%)	11 (22%)	17 (17%)
Complications during admission	79 (52%)	36 (71%)	43 (43%)
Cerebral arterial vasospasm	45 (30%)	20 (39%)	25 (25%)
Ventriculitis	11 (7%)	10 (20%)	1 (1%)
Hydrocephalus	58 (38%)	30 (50%)	28 (28%)
Discharged to same setting as at admission	95 (63%)	9 (18%)	86 (85%)

Loan JJM, *et al. Stroke Vasc Neurol* 2023; 8:e001583. doi: 10.1136/svn-2022-001583

Supplementary figure 1: Exploratory analyses treatment and duration of hyponatraemia.

Bar chart of prescription of supplementary sodium (A) and scatter plots of percentage of each patient's hyponatraemic period that a low or neutral fluid balance was achieved for all hyponatraemic patients (B) and those with moderate-severe hyponatraemia (C).

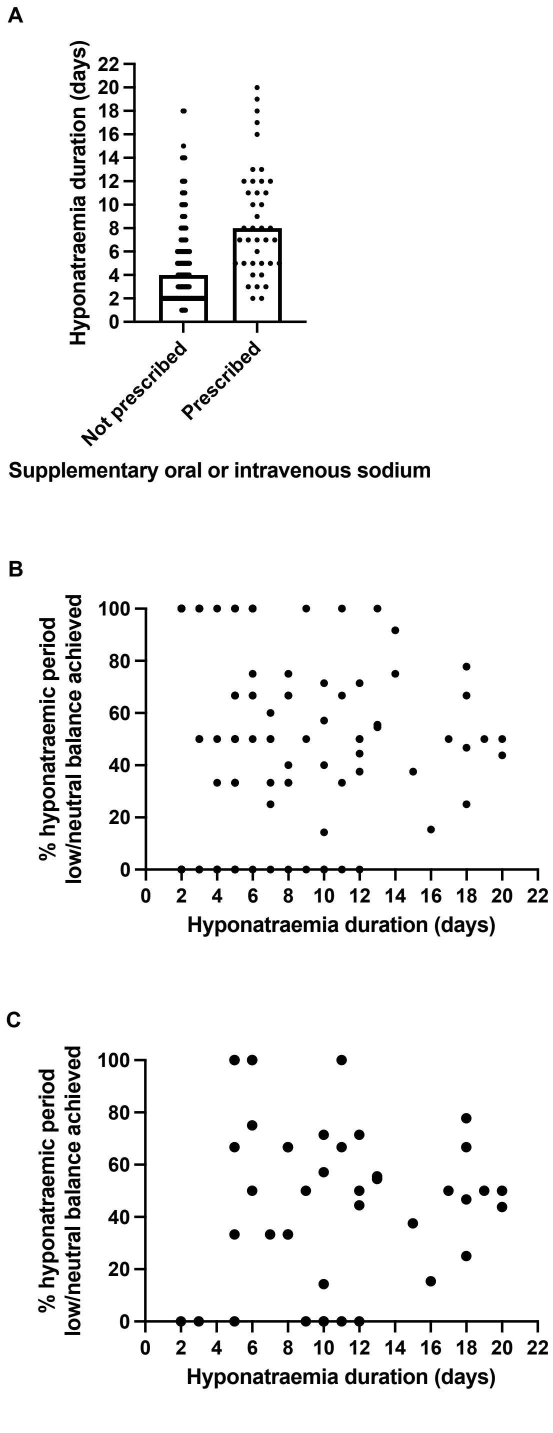

Supplement: Supplementary data [file svn-2022-001583supp001.pdf]
